# Supplementary material for: Checkpoint blockade and nanosonosensitizer-augmented noninvasive sonodynamic therapy combination reduces tumour growth and metastases in mice
Source: Nat Commun. 2019 May 2;10:2025. doi: 10.1038/s41467-019-09760-3 (PMC6497709; doi:10.1038/s41467-019-09760-3)
Supplement: Supplementary file 1 — Supplementary Information [file 41467_2019_9760_MOESM1_ESM.pdf]

Supporting Information

Checkpoint blockade and nanosonosensitizer-augmented noninvasive sonodynamic therapy combination reduces tumour growth and metastases in mice

Yue et al

Supplementary Figures

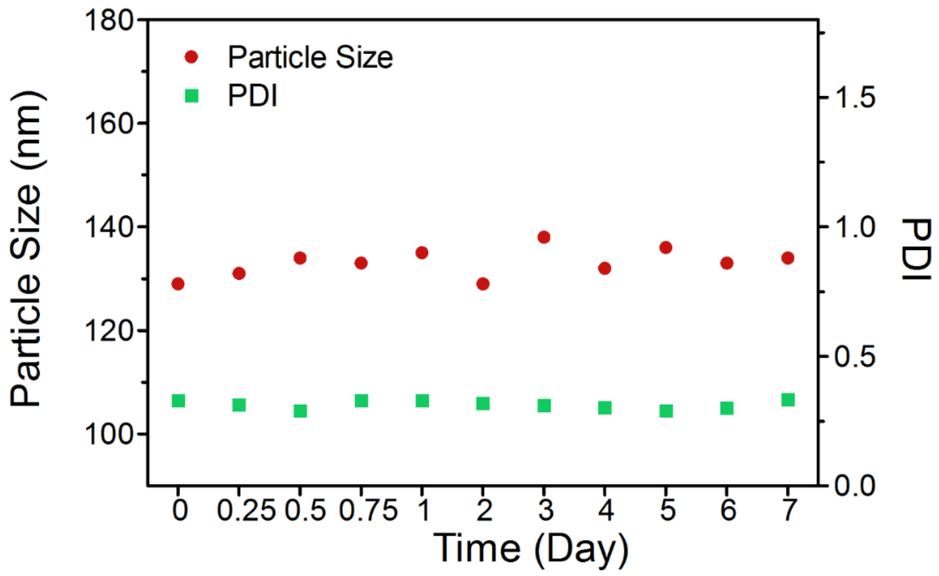

Supplementary Figure 1. Time-dependent particle size and polydispersity index (PDI) changes of HMME/R837@Lip in PBS at 4°C.

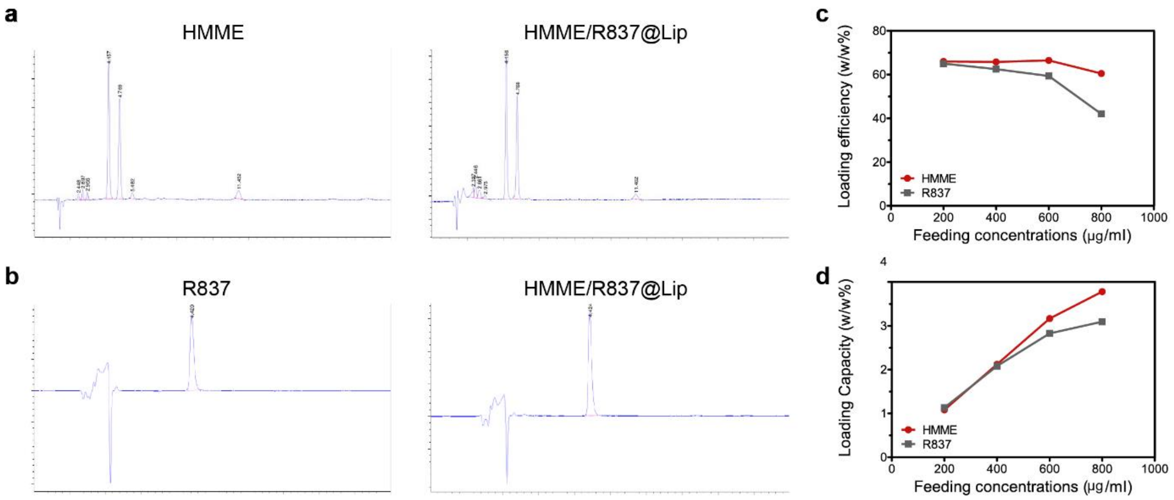

Supplementary Figure 2. High pressure liquid chromatograph (HPLC) absorbance of HMME (a), R837 (b) and HMME/R837@Lip, indicating the successful co-loading of HMME and R837 into Lip; (c, d) The loading efficiency (c) and loading capacity (d) of HMME and R837 by Lip nanoparticles obtained at different feeding concentrations as measured by HPLC.

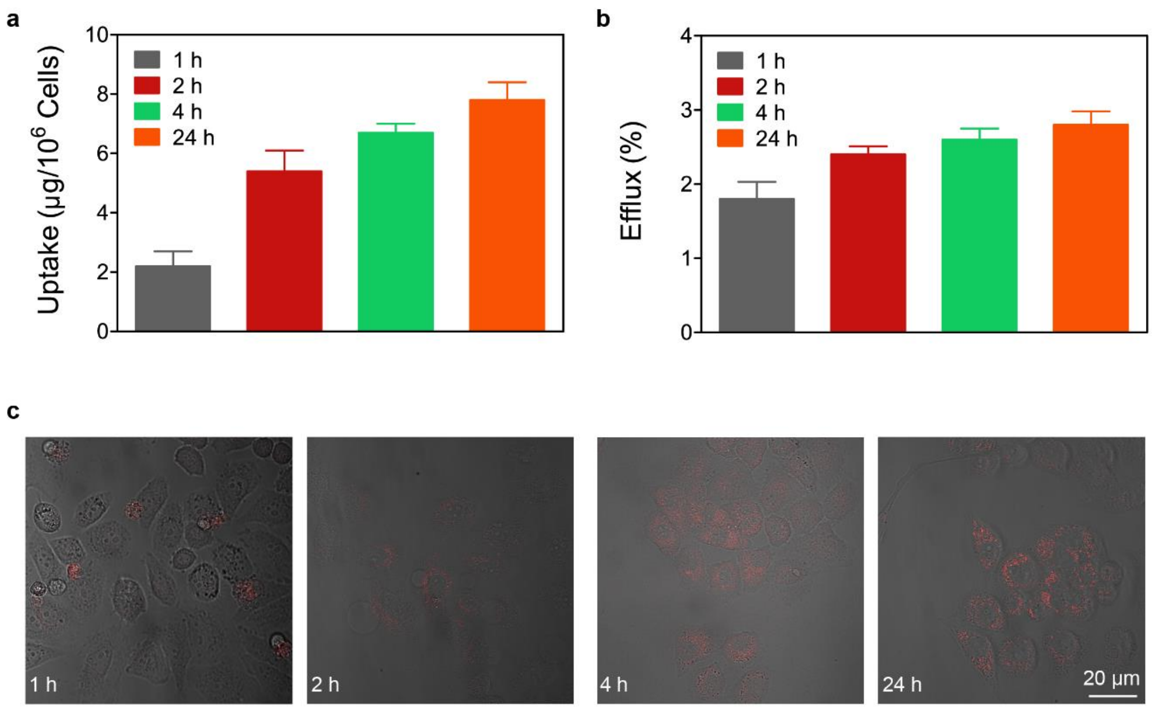

Supplementary Figure 3. (a) Time-dependent cellular uptake of HMME/R837@Lip in 4T1 cells; Error bars are based on SD (n = 3); (b) Efflux of HMME/R837@Lip by 4T1 cells; Error bars are based on SD (n = 3); (c) Confocal imaging showing the time-dependent cellular uptake of HMME/R837@Lip in 4T1 cells (scale bar = 20 μm).

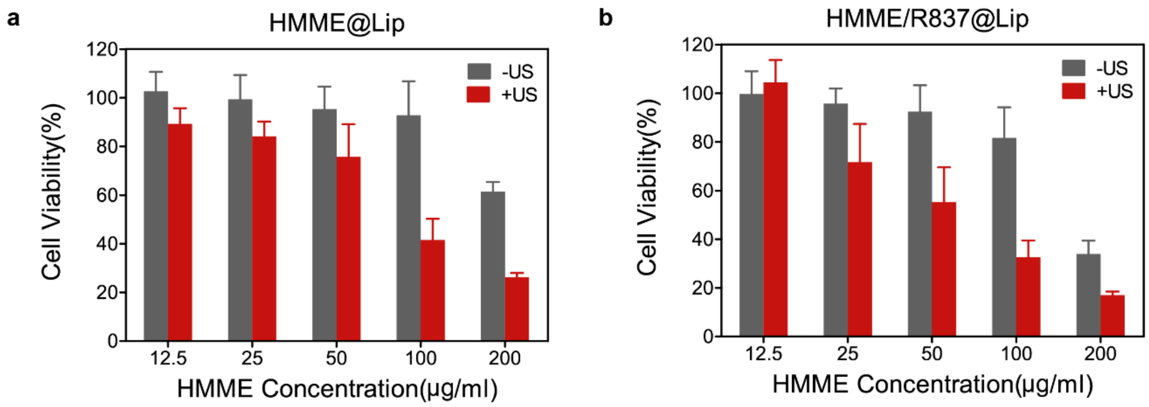

Supplementary Figure 4. Cell viabilities of 4T1 cells after co-incubation with (a) HMME@Lip or (b) HMME/R837@Lip for 24 h and exposed to US irradiation at varied concentrations. (US, 1.0 MHz, 1.5 W/cm^2, 50% duty cycle). Error bars are based on SD (n = 5).

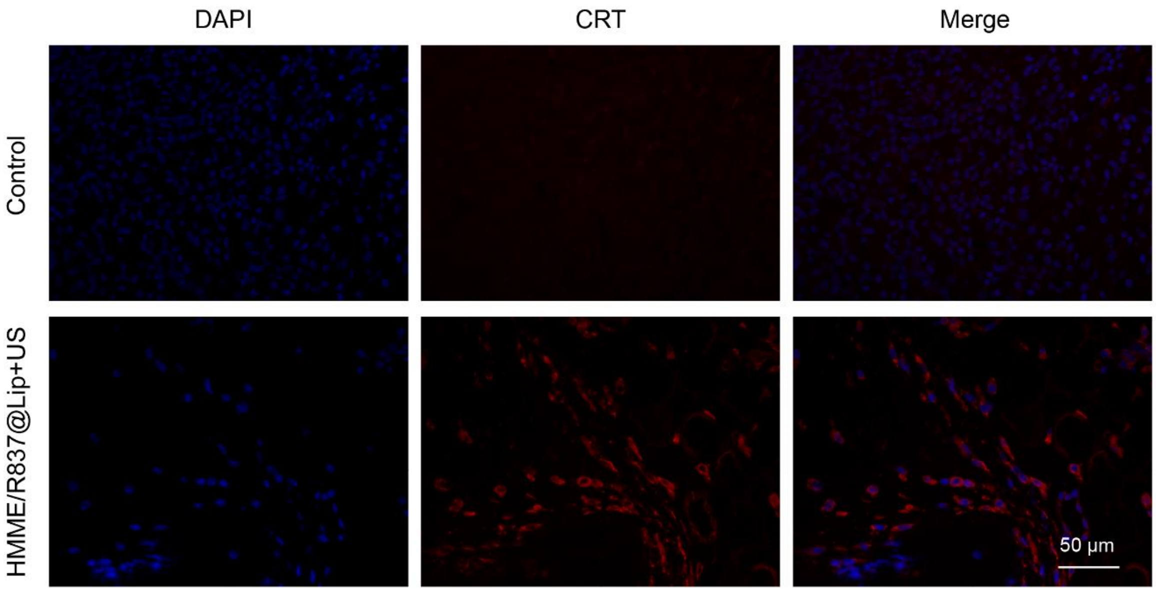

Supplementary Figure 5. Representative CLSM images showing the CRT exposure on 4T1 tumour cells after treatment with HMME/R837@Lip plus ultrasound irradiation (scale bar = 50μm).

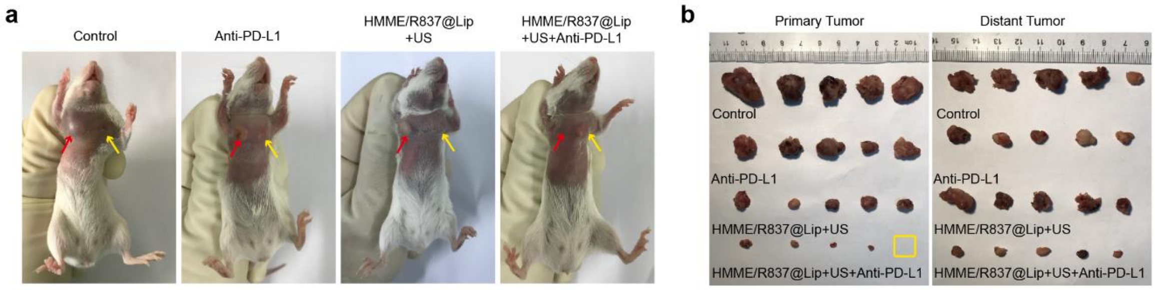

Supplementary Figure 6. (a) Photographs of the 4T1-tumor bearing mice and the tumor regions at the end point; (b) Photographs of excised primary and distant tumors at the end point. Red arrows indicate primary tumours and yellow arrows indicate mimic distant tumours.

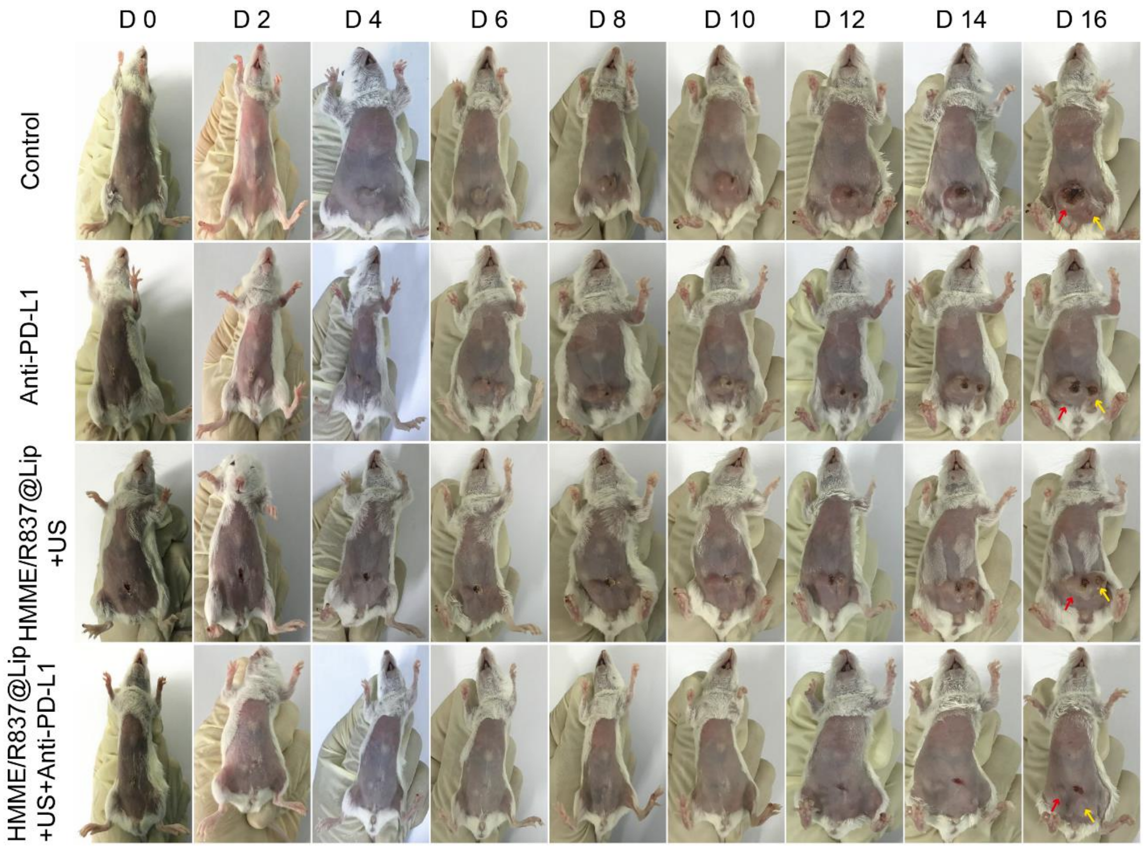

Supplementary Figure 7. Photographs of 4T1 orthotopic tumor -bearing mice at different periods after different treatments. Red arrows indicate primary tumours and yellow arrows indicate mimic distant tumours.

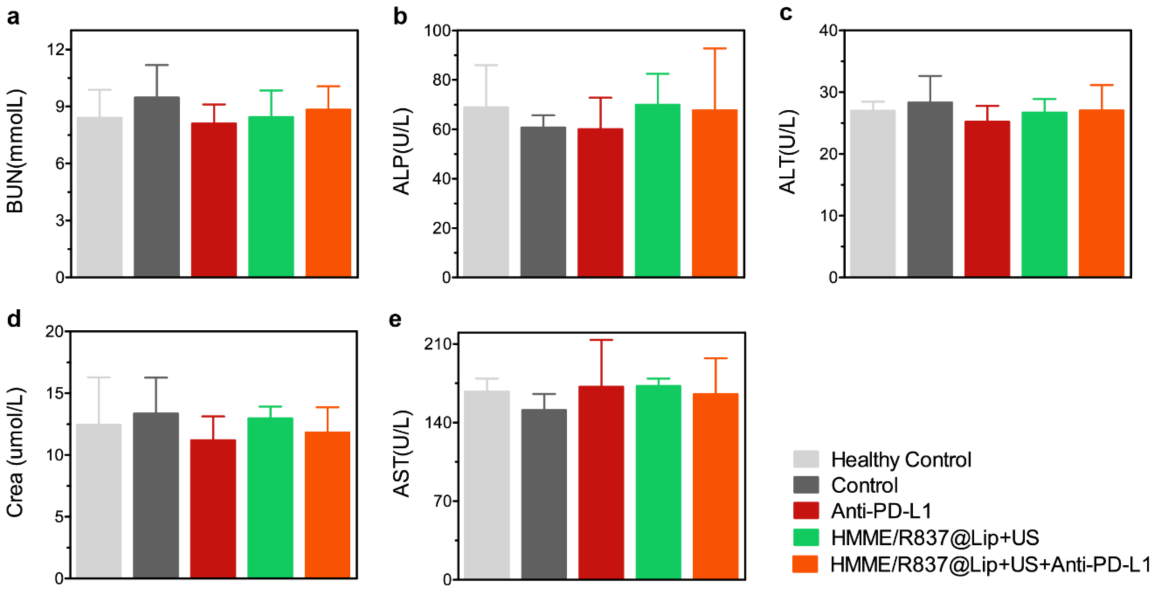

Supplementary Figure 8. Serum biochemistry data. Balb/c mice bearing 4T1 tumor sacrificed at 12 days after treatments. Untreated healthy mice were used as the control. Serum biochemistry data including Blood urea nitrogen (BUN) (a), Alkaline phosphatase (ALP) (b), Alanine aminotransferase (ALT) (c), Creatinine (d) levels and Aspartate aminotransferase (AST) (e) were measured. Error bars are based on SD (n = 3).

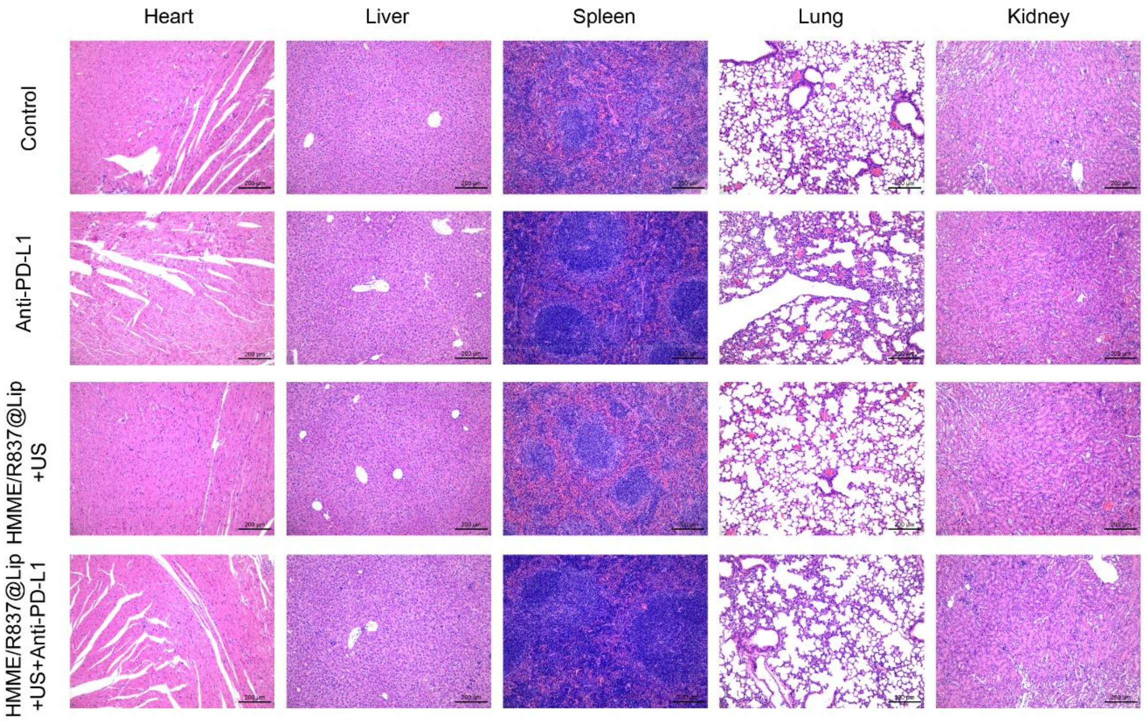

Supplementary Figure 9. H&E-stained tissue sections of major organs (heart, liver, spleen, lung and kidney) from mice with different treatments (All scale bar = 200 μm).

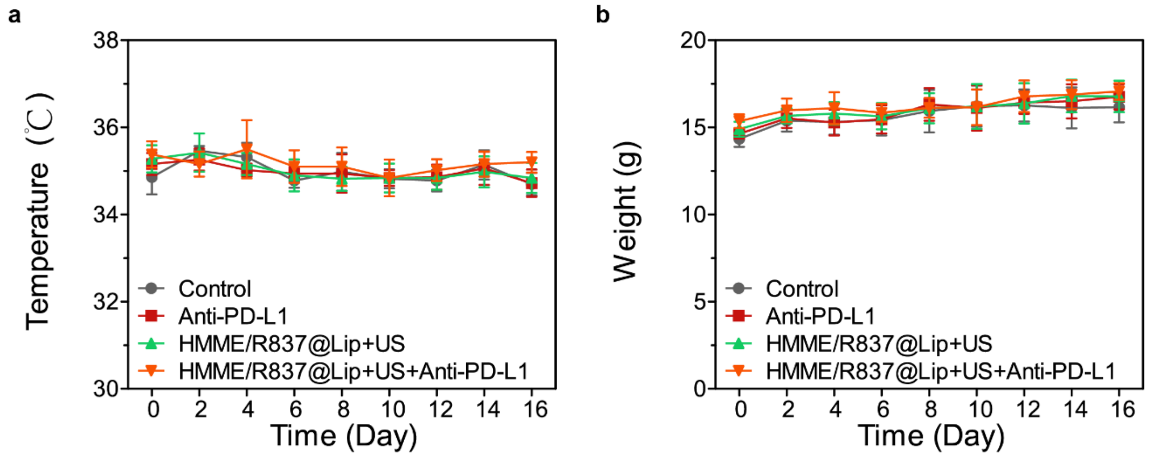

Supplementary Figure 10. Time-dependent body temperature (a) and weight (b) surveillance of mice (n = 5, mean ±standard deviation) after different treatments with the 4T1 orthotopic tumor models.

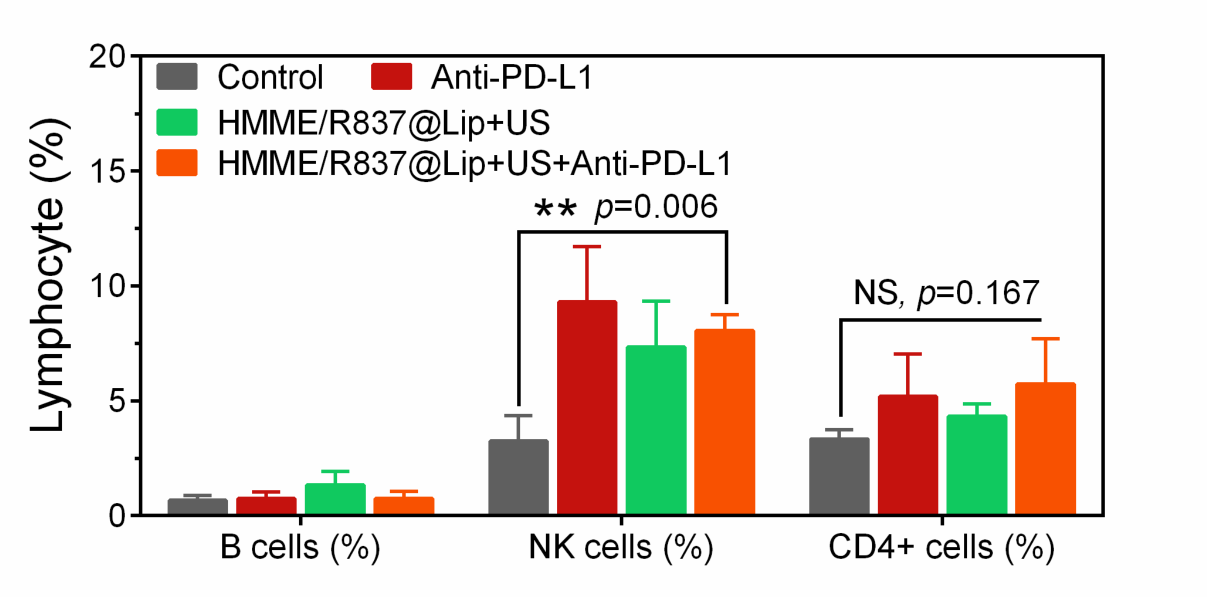

`

Supplementary Figure 11. Absolute quantification analysis of the tumor infiltrating leukocytes cells including B cells ($CD45^+CD3e^-B220^+$), NK cells ($CD45^+CD3e^-NKp46^+$) and $CD4^+$ T cells ($CD45^+CD3e^+CD4^+$) in distant tumors by flow cytometry. Error bars are based on SD (n = 3). Statistical significances were calculated *via* Student's *t*-test; NS, not significant; ** $p < 0.01$.

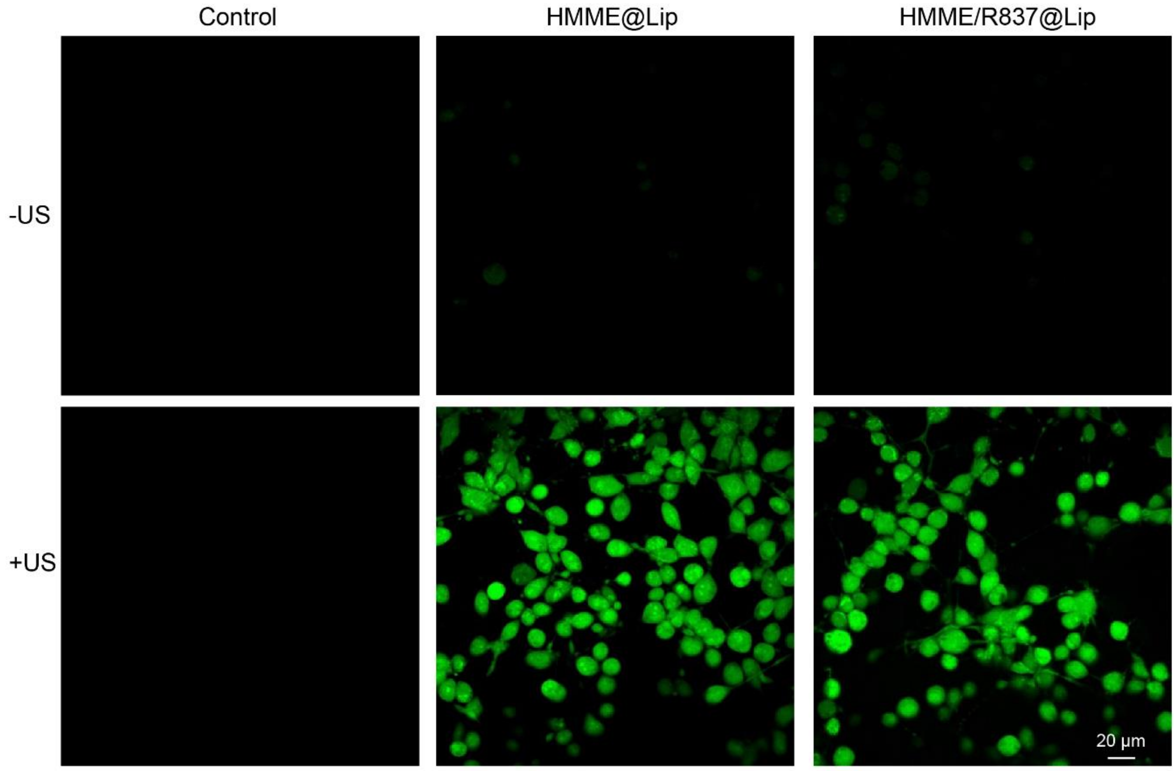

Supplementary Figure 12. CLSM images of CT26 cells stained with DCFH-DA after different treatments: control (without any treatment), HMME@Lip only, HMME/R837@Lip only, US only, HMME@Lip combined with US irradiation, HMME/R837@Lip combined with US irradiation (scale bar =20 μm).

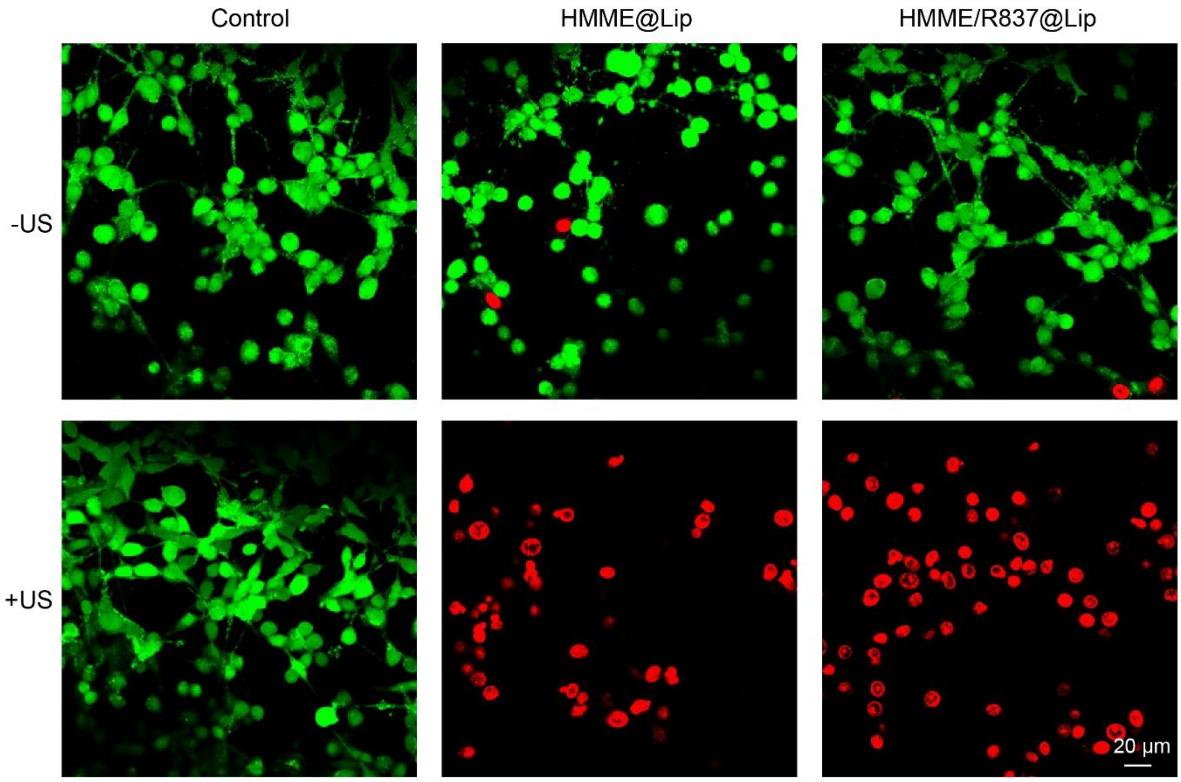

Supplementary Figure 13. CLSM images of CT26 cells stained with Calcein AM and PI after various treatments: control (without any treatment), HMME@Lip only, HMME/R837@Lip only, US only, HMME@Lip combined with US irradiation, HMME/R837@Lip combined with US irradiation (scale bar = 20 μm).

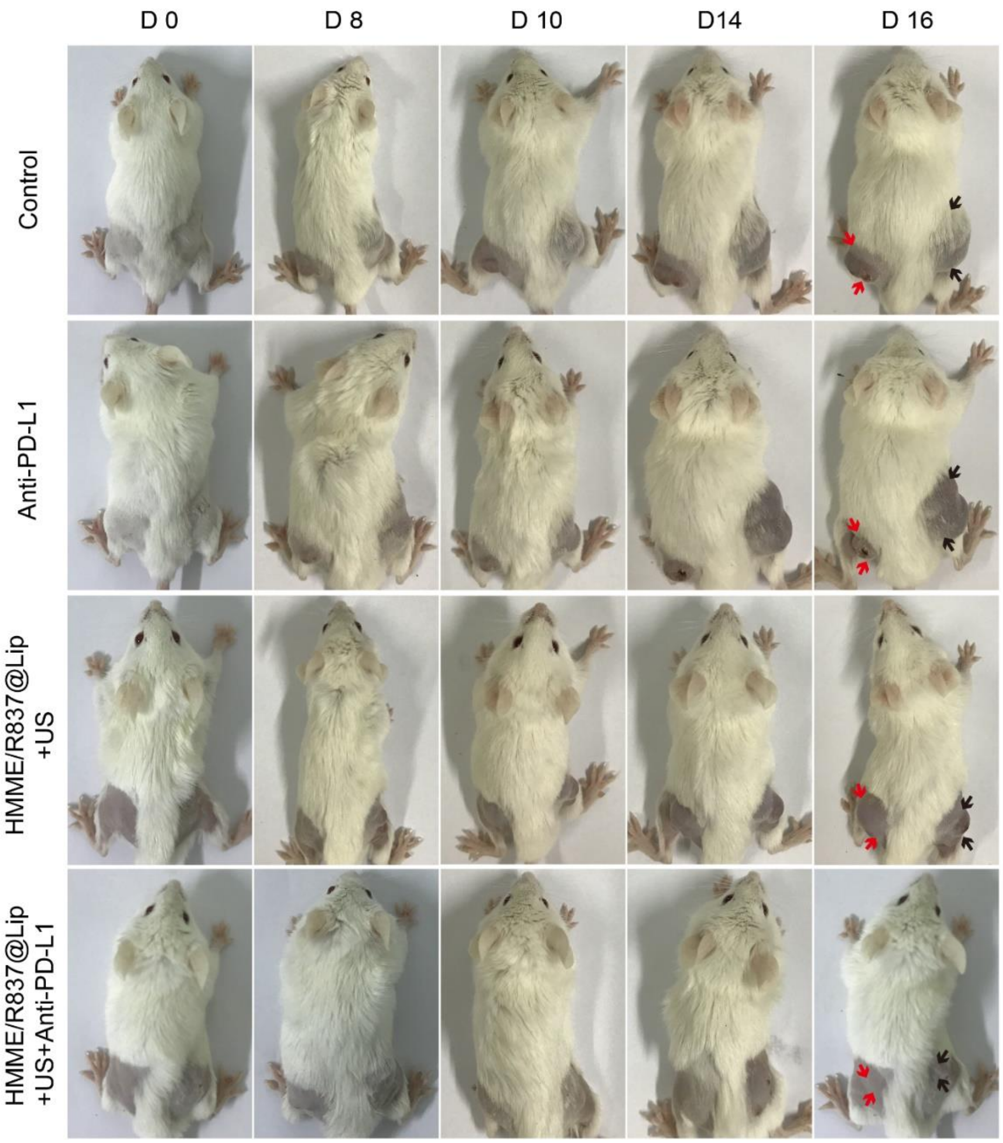

Supplementary Figure 14. Photographs of CT26 tumor -bearing mice at different periods after different treatments. Black arrows indicate primary tumours, and red arrows indicate mimic distant tumours.

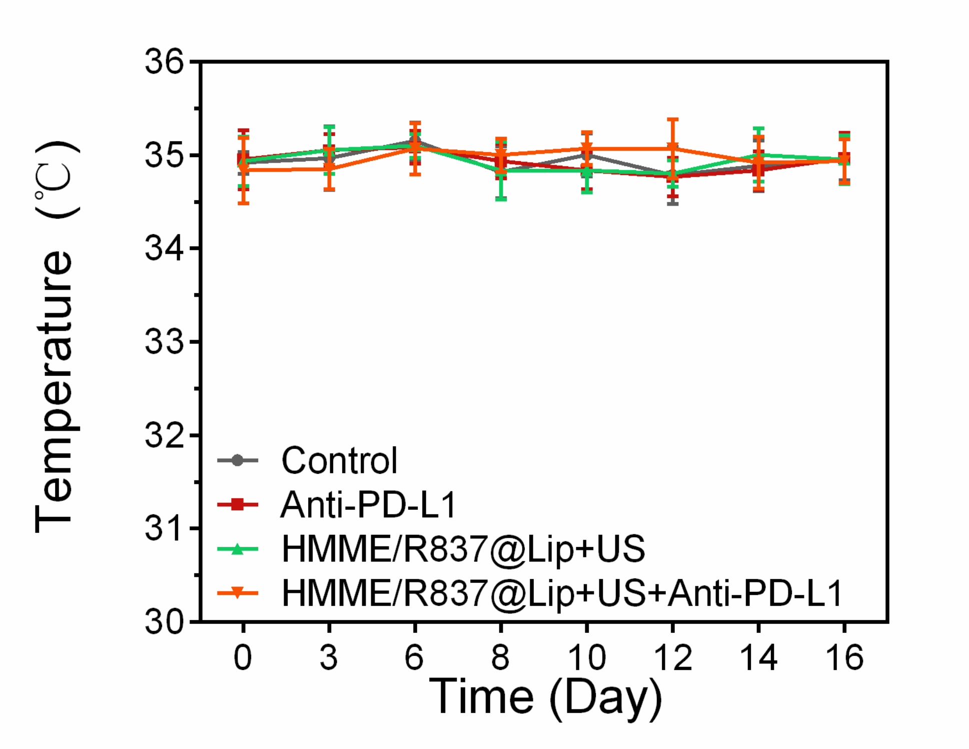

Supplementary Figure 15. Time-dependent body-temperature surveillance of CT26 tumor-bearing mice (n = 6, mean ± standard deviation) after different treatments.

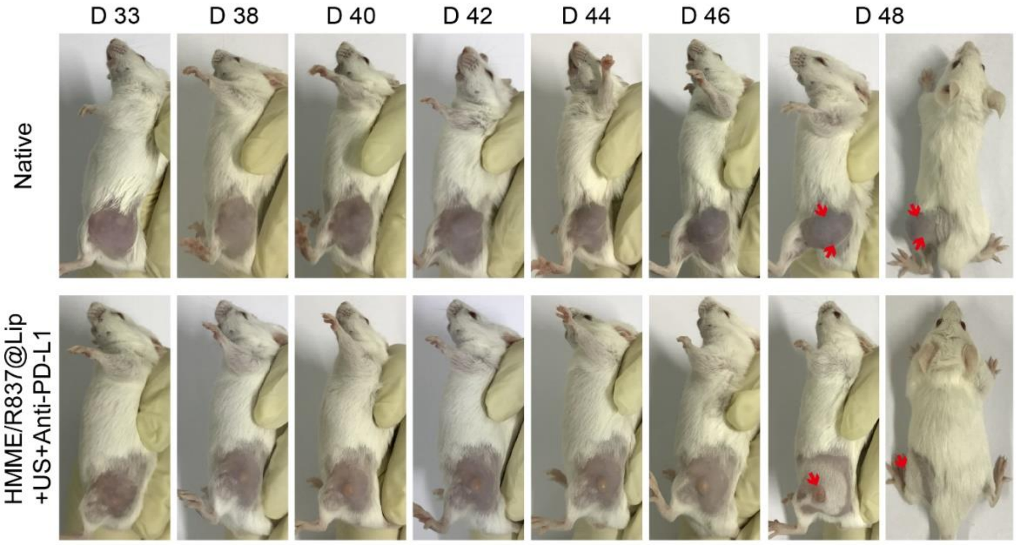

Supplementary Figure 16. Photographs of CT26 tumor -bearing mice (the rechallenged tumours, bottom) at different periods after the combined treatment (HMME/R837@Lip-augmented SDT plus anti-PD-L1). The age- and sex-matched native mice were inoculated with the same number of CT26 cells to help to identify the treatment results (above); Red arrows indicate the rechallenged tumours.
